# Recurrent Spontaneous Coronary Artery Dissection: Association with Takotsubo Syndrome and Fibromuscular Dysplasia; Comprehensive Review

**DOI:** 10.31083/j.rcm2311367

**Published:** 2022-10-27

**Authors:** Shams Y-Hassan, Goran Abdula, Felix Böhm

**Affiliations:** ^1^Coronary Artery Disease Area, Heart and Vascular Theme, Karolinska Institutet and Karolinska University Hospital, 14152 Stockholm, Sweden; ^2^Department of Clinical Physiology, Karolinska University Hospital and Karolinska Institutet, 14152 Stockholm, Sweden; ^3^Department of Cardiology, Danderyd Hospital and Karolinska Institutet, 18288 Danderyd, Sweden

**Keywords:** spontaneous coronary artery dissection, takotsubo syndrome, fibromuscular dysplasia, myocardial infarction, recurrent SCAD

## Abstract

Spontaneous coronary artery dissection (SCAD) is a non-traumatic, non-iatrogenic, and non-atherosclerotic separation or dissection of the coronary arterial wall by the formation of an intramural hematoma causing a false lumen leading to compression of the true lumen with a varying degree of coronary blood flow obstruction. One of the important and frequent complications of the disease is the in-hospital and long-term SCAD recurrence. SCAD associated with takotsubo syndrome (TS) has been described in case reports, series of cases and in some studies. Some investigators believe that the association of SCAD and TS is a misdiagnosis. The association of SCAD and fibromuscular dysplasia (FMD) has received major attention during the last 10 years. In this report, the short and long-term SCAD recurrence, SCAD association with TS and FMD are reviewed and demonstrated with illustrative images.

## 1. Introduction

Spontaneous coronary artery dissection (SCAD) is a non-iatrogenic, non-traumatic, and non-atherosclerotic separation or dissection of the coronary arterial wall by the formation of an intramural hematoma (IMH), with or without intimal tear, creating a false lumen compressing the true lumen of the artery and causing a varying degree of coronary blood flow obstruction [1, 2]. The proposed pathophysiological mechanisms of SCAD are acute hemorrhage in the coronary artery wall caused either by a primary intimal tear or acute hemorrhage within the tunica media due to spontaneous rupture from the increased density of the vasa vasorum [1, 2, 3]. Worth to mention, in a study with optical coherence tomography (OCT) imaging, Jackson *et al*. [4] reported no significant differences in the vasa vasorum density between SCAD cases and control non-SCAD myocardial infarction (MI) cases. Almost 90% of SCAD patients are women with a mean age of 45 to 52 years [3]. SCAD is frequently preceded by an emotional or a physical stressor [3]. The disease may be precipitated by recreational drugs, intense physical exercise, or heavy isometric activity [1, 2, 3]. In almost 90% of cases, SCAD presents with the manifestation of acute coronary syndrome (ACS), which may be in the form of ST-elevation myocardial infarction (STEMI) or non-STEMI (NSTEMI) [1, 2]. The proportion of STEMI presentation has varied in different studies, ranging from 24% to 87% [5]. In less than 10%, SCAD may present with ventricular arrhythmias, cardiogenic shock, or rarely sudden death. In sudden cardiac death of relatively young patients, especially women, one should look closely at the coronary arteries for SCAD, which can be missed at autopsy [6].

The most important diagnostic procedure to identify SCAD is the invasive coronary angiography (CAG). SCAD is classified simply into angiographically visible SCAD, which corresponds to type 1 SCAD lesions according to Saw classification [7, 8] and angiographically invisible SCAD, which corresponds to type 2A, 2B and type 3 according to Saw classification [7]. In angiographically visible SCAD, the pathognomonic angiographic signs of a radiolucent intimal flap, or contrast staining of the vessel wall, and double or multiple radiolucent lumens of different opacities, are seen during invasive CAG [8]. In the angiographically invisible SCAD, the mentioned pathognomonic signs are not seen, but certain other features may raise the suspicion of SCAD. This could be a long diffuse smooth narrowing of the coronary artery with abrupt demarcation of the proximal normal part of the vessel and a normal segment after the end of the lesion (type 2A) [8]. The angiographically invisible SCAD may also involve the peripheral segments of the coronary arteries and be seen as “normal tapering” vessel (type 2B) [7, 8]. The lesions in the angiographically invisible SCAD may also be short mimicking an atherosclerotic coronary artery lesion (type 3) [7, 8]. With invasive CAG, the diagnosis of angiographically invisible SCAD will be suspected and this may be confirmed by invasive intra-coronary imaging such as intravascular ultrasound (IVUS) and/or OCT [8, 9, 10, 11], where an intimal tear and/or an IMH and double lumen (false and true) will confirm the SCAD diagnosis. The combination of characteristic angiographic signs mentioned above, and OCT imaging facilitates the diagnosis of ambiguous SCAD cases without intimal rupture [12]. When SCAD lesions are located in the peripheral coronary arteries and therefore intravascular imaging deemed to be associated with substantial risks, the diagnosis of the angiographically invisible SCAD may be confirmed by repeated coronary angiography after 6–8 weeks where angiographic healing of the SCAD lesions is seen [8].

One important and frequent complication of the disease is the in-hospital and long-term SCAD recurrence [1, 2, 3]. The increase in knowledge and recognition of the disease has resulted in novel information that may affect the future management of these patients. Coexistence of SCAD and Takotsubo syndrome (TS) in the same patients has been reported in case reports [13, 14, 15, 16, 17, 18, 19, 20], series of cases [21] and in some studies [22]. Some investigators still believe that the association of SCAD and TS is a misdiagnosis [23]. The association of fibromuscular dysplasia (FMD) and SCAD has received major attention during the last 10 years [1, 2, 3]. In this report, the short- and long-term SCAD recurrence, SCAD association with TS and FMD are reviewed comprehensively and demonstrated with illustrative images.

## 2. Early and Late Recurrent SCAD 

**Fig. 1.**
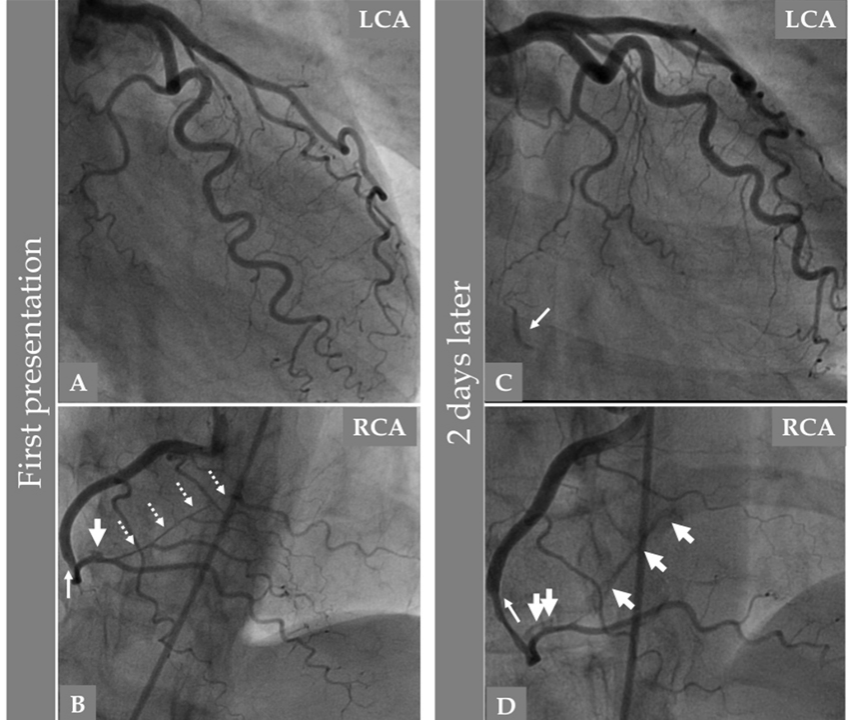
**Invasive left and right coronary angiography of a 54-year-old female patient presented with chest pain while walking home after work (A and B) and 2 days later because of repeated chest pain (C and D)**. Apart from the tortuous Left coronary artery (LCA), the artery and its branches are normal (A). The right coronary artery (RCA) angiography shows a narrowing which begins above the acute marginal region down to the bifurcation of posterior descending artery (PDA)/posterior left ventricular artery (PLVA) (B thin white arrow) and then a long tight tubular stenosis of the PLVA (B, broken white arrows). Before the PDA/PLVA bifurcation a spot of black contrast hold-up in the vessel wall is seen (B, thick white arrow), these findings together indicate spontaneous coronary artery dissection (SCAD) in the RCA. Two days later, the LCA angiography shows unchanged LCA apart from the appearance of collateral circulation to a segment of the PLVA (C, white arrow). The RCA angiography shows extensive SCAD progression with proximal extension of intramural hematoma (IMH) (D, thin white arrow) and distal extension of IMH leading to occlusion of the PLVA with contrast staining of the vessel wall in a long segment of the PLVA (D, thick white arrows). Contrast hold-up before the PDA/PLVA bifurcation is increased (D, two thick white arrows). These findings indicate an extension of the SCAD in both antegrade and retrograde directions.

**Fig. 2.**
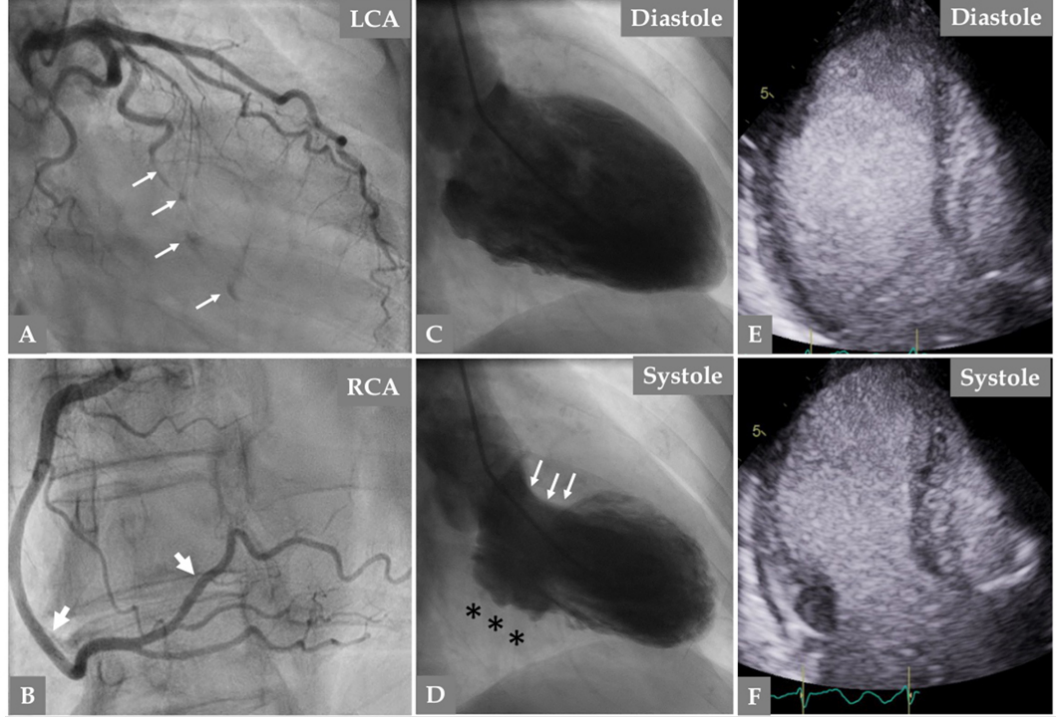
**Urgent invasive coronary angiography because of new chest pain while watching TV in the same patient (at the age of 66 years) in Fig. 1, twelve years after the first SCAD episode, reveals normal left main stem and left anterior descending artery (LAD)**. (A) The obtuse-marginal (OM) branch is occluded with spots of contrast in the path of the occluded OM branch (A, thin white arrows). Right coronary artery angiography reveals complete resolution of the previously dissected segments of RCA (B thick white arrows). Contrast Left ventriculography during diastole (C) and systole (D) reveals akinesia in the inferior basal segment (D, black asterisks) but good contractions in the anterior basal segment (D, white arrows). There is mid-apical ballooning with apical tip sparing (D), a finding consistent with mid-apical Takotsubo syndrome (TS). The mid-apical ballooning pattern was confirmed with contrast echocardiography (E, diastole and F, systole). These findings together suggest recurrent SCAD in a new coronary artery associated with TS, and complete resolution of the previous SCAD in the RCA.

SCAD is characterized by a relatively high recurrence rate, which may occur both early and late in the course of the disease. The frequency of recurrent SCAD varies widely in the literature and has been reported to occur in 5 to 29% of SCAD patients [1, 2, 24]. Recurrence of SCAD during the acute and subacute stages of the disease may either be attributed to the worsening or extension of the index lesion in the proximal or distal direction (Fig. 1), or it may be due de novo SCAD occurring distinct from the index lesion. After one month from the index lesion, SCAD recurrence usually occurs in a new previously unaffected coronary segment and recurrence may occur up to 15–20 years after the index presentation [24] (Fig. 2). Waterbury and colleagues [25] reported significant SCAD progression in 42 of 240 (17.5%) SCAD patients after attempted initial conservative therapy and 91% of the SCAD progression occurred during the first 6 days after admission. The IMH was shown to progress in both proximal and distal directions to the initial lesion [25]. SCAD progression demonstrated in Fig. 1 occurred 2 days after the index SCAD and progressed both proximally and distally (Fig. 1A–D). SCAD patients with an IMH alone usually demonstrate significantly more proximal lesion propagation than those with intimal tear [25]. The main predictors for SCAD progression in that study were isolated IMH, multivessel SCAD, severe lesions with stenosis grade >80%, lesion length >60 mm, and SCAD lesions in the left anterior descending artery (LAD) [25]. Main *et al*. [26] have also reported on early SCAD extension and late de novo SCAD recurrence by studying 43 patients with recurrent SCAD. Nine (20.9%) of the 43 patients had extension of SCAD at a median time of five (1–19) days from index presentation. All SCAD extension patients had worsening of the index SCAD lesions with 5/9 involving extension to the adjacent segments. Thirty-four (79.1%) of the 43 patients had de novo recurrent SCAD at a median time of 1487 (107–6461) days after the SCAD admission. All de novo recurrent SCAD events affected a new segment distinct from the index dissection as demonstrated in Fig. 2.

Many patients express concerns about the recurrence of SCAD. Several factors predisposing the onset and recurrence have been discussed. Important predisposing factors are hypertension, migraine headache, inherited connective tissue diseases, pregnancy, coronary tortuosity, and FMD [1, 2, 3, 24]. It is well-known that the traditional cardiovascular risk factors reported in atherosclerotic coronary artery diseases such as smoking, and hyperlipidemia are less frequent in patients with SCAD. However, hypertension has been reported to predispose recurrent SCAD [27]. In a prospective study of 327 SCAD patients with a median follow-up of 3.1 years, recurrent SCAD occurred in 10.4% of patients [27]. In multivariate modeling, hypertension was significantly associated with increased risk of SCAD recurrence (hazard ratio: 2.46; *p *< 0.011) [27]. In the same study, reduced recurrence rate occurred in patients with beta blocker therapy (hazard ratio: 0.36; *p *< 0.004) [27]. Rigatelli and colleagues [28] also reported in a small study that hypertension was more frequent in patients with SCAD recurrence (50% vs 13%, *p* = 0.04). In the same study comparing 31 patients with a single SCAD episode and 6 patients with recurrent SCAD, the following factors were associated with SCAD recurrence: arterial hypertension, a string-like sign measuring >15 mm on CAG, and IMH on IVUS imaging measuring >25 mm [28]. The time between the first and the second SCAD events was 58.8 ± 27.9 months [28]. A history of migraine headache has been reported in about one third of patients with SCAD [24]. Among 585 SCAD patients 236 (40%) had a history of migraine. At 5 years, however there was no difference in SCAD recurrence for those with versus without migraine (15% vs 19%; *p* = 0.39) [29]. The relationship of connective tissue diseases (Ehlers-Danlos syndrome, Marfans syndrome, and Loeys-Dietz syndrome) as a predictor of SCAD recurrence is indistinct [24].

In a study of 246 patients with SCAD, Eleid *et al*. [30] reported that 78% of SCAD patients had coronary artery tortuosity compared to 17% of control patients. Recurrent SCAD was associated with severe coronary artery tortuosity [24, 30]. The recurrence rate of SCAD was higher among patients with FMD in a systematic review of 24 observational studies constituting a total of 1720 patients [31]. Coronary artery tortuosity is clearly seen in the obtuso-marginal branch (OM) in Fig. 1 and recurrent SCAD occurred in this branch as seen in Fig. 2.

Among the therapeutic agents, treatment with beta blockers was associated with reduced risk of recurrent SCAD in a study of 327 patients with SCAD [27]. Other medications prescribed for patients with SCAD, as calcium channel blockers, aspirin and other antiplatelet drugs, have not been found to have any association with SCAD recurrence [24].

## 3. Association of SCAD and Takotsubo Syndrome

TS, also called neurogenic stunned myocardium or broken heart syndrome, is an acute cardiac disease entity, which presents with a clinical picture indistinguishable from that of an ACS and characterized by a transient left ventricular (may also be right) myocardial stunning [32, 33]. The term “Tsubo” or “Takotsubo” was introduced in the early 1990s by Sato and Dote to describe the silhouette of the left ventricle during systole in patients presenting with a clinical picture of MI with no obstructive coronary artery disease [34, 35]. The defining feature of TS is the regional left ventricular wall motion abnormality (LVWMA) with a unique circumferential pattern resulting in a ballooning of the left ventricle during systole [32, 33, 35]. The LVWMA in TS is incongruent with the coronary artery supply territories and is reversible with almost complete resolution of ventricular dysfunction in hours to weeks [32]. The left ventricular ballooning pattern may be apical, mid-ventricular, basal, or focal [32, 33]. A global left ventricular contractile abnormality has also been reported [36]. The right ventricle is involved in about 30% of TS cases [37]. A trigger factor (emotional or physical) may precede the onset of TS in about 70% of cases [33, 38]. Innumerable physical triggers, extending from serious diseases such as intracranial hemorrhage, sepsis to the most physiological processes as sexual intercourse, may trigger the syndrome [38, 39]. One of the important and currently accepted and well-documented trigger factors is the ischemic insult caused by ACS including SCAD [14, 21, 22, 40, 41, 42].

SCAD and TS have many features in common. TS may present with features resembling that of an ACS including SCAD. Some researchers include TS as a common non-atherosclerotic cause of ACS [43], others believe that TS is not an ACS [44] but TS may be triggered by an ACS [40] or complicated by an acute MI [45]. Both TS and SCAD affect predominantly women, and both may be preceded by an emotional or a physical stress factor. Durable and intense emotional stress (usually in women) and an intense isometric physical exercise (usually in men) are the most reported precipitants of SCAD [1]. Both conditions are characterized by recovery of the pathological condition; “restitution ad integrum”, which implies angiographic healing of the dissected vessel as a rule in SCAD and recovery of left ventricular dysfunction within weeks in TS [5, 32]. Compared to TS, patients with SCAD are significantly younger. Patients with TS have worse in-hospital complications and outcomes including a higher risk of mortality during the index admission compared with SCAD [43]. Cammann* et al*. [46] reported that left ventricular ejection was more impaired in TS compared to SCAD. In addition, 30-day mortality was significantly higher in TS patients in the same study. On the contrary, the recurrence rate is higher both during the admission days and follow up in SCAD compared with TS [1, 2, 33, 47]. After a median follow-up of 5 years, Macaya *et al*. [48] reported that the diagnosis of SCAD compared to TS conferred a significantly worse clinical outcome. This was mainly driven by rehospitalization for cardiac causes.

Coexistence of SCAD and TS in the same patients have been reported in case reports [13, 14, 15, 16, 17, 18, 19, 20], series of cases [21] and in some studies [22]. These associations have been deemed as SCAD misdiagnosed as TS by some authors [23] or as TS triggered by SCAD by other investigators [8, 21, 22, 41]. The first report on the association of SCAD and TS was published in 2013 in a woman with LAD-SCAD with mid-apical ballooning where the reversible myocardial stunning extended beyond the limited MI caused by LAD-SCAD [13]. During the last few years, series of cases, and even some studies of ACS including SCAD triggering TS have been reported [13, 14, 15, 16, 17, 18, 19, 20]. Duran *et al*. [22] reported in one study a high prevalence of SCAD and concomitant TS. Among 43 patients with SCAD who underwent contrast left ventriculography at the time of coronary angiography, 24 (56%) had LVWMA consistent with TS. The authors concluded that TS may be a plausible cause for SCAD. Salamanca *et al*. [43] performed left ventriculography in 72 (23%) out of 318 patients with SCAD. Fourteen (19%) of 72 patients had TS-like apical ballooning pattern on the left ventricular angiogram. The most reasonable proposed mechanism of SCAD inducing TS is through the acute ischemic insult and the intense physical discomfort as chest pain caused by SCAD may act as an intense physical stressor [21, 32, 49, 50, 51, 52].

On careful review of the literature, one may find case reports where the association of SCAD and TS may be suspected with justifiable reasons, but SCAD was either missed or misdiagnosed as another coronary lesion [21, 53, 54]. During the last years, several other publications reported that SCAD was either “misdiagnosed as TS” [23, 55] or TS was excluded or deemed to be misleading when the SCAD diagnosis was confirmed [56, 57]. Chou *et al*. [23] reported on 9 cases of “SCAD misdiagnosed as TS”. The authors deemed that the LVWMA in these 9 patients were congruent to the territories supplied by the dissected coronary arteries. Furthermore, the investigators deemed that troponin elevation were too high to be interpreted as a result of TS [58]. This study and the disagreement on the presence of an association between SCAD and TS has been discussed previously; we believe that the SCAD was missed and not misdiagnosed as TS and the 9 cases reported by Chou and colleagues had SCAD and TS concurrently [58, 59, 60]. Similar thoughts on the existence of an association of TS and SCAD have been presented by other investigators [61].

Furthermore, after the report of Chou and colleagues on the 9 cases of “SCAD misdiagnosed TS”, the same investigators have reported on LVWMA in a relatively large numbers of patients with SCAD in several reports [62, 63, 64]. Franco *et al*. [62] reported on the LVWMA in 85.6% of 277 patients with SCAD. The LVWMA were described as hypokinesis, akinesis, or dyskinesis corresponding, according to the authors, to the dissected coronary arteries. In 26.0% of cases, the left ventricular ejection fraction (LVEF) was <50%; in 5.1%, there was moderate to severe LV dysfunction with LVEF <40%. The authors [62] reported improvement of left ventricular function during follow up and acknowledged that the improvement in the left ventricular function may reflect resolution of myocardial stunning. The authors hypothesized that the SCAD could result in prolonged myocardial ischemia and potentially causing myocardial stunning to a greater degree than myocardial infarction but insisted that the segments with LVWMA were subtended by the dissected coronary artery. Similar pattern of LVWMA have been reported in a large multicenter Canadian SCAD cohort study of 750 patients with SCAD [63], and in another study of 25 SCAD vessels in 22 patients [64]. One could expect that the largest number of patients with concurrent SCAD and TS should be reported in the Vancouver SCAD studies, but surprisingly, myocardial stunning with a pattern of TS in the above-mentioned large studies were not mentioned [62, 63]. The notion that SCAD has been misdiagnosed as TS is even reflected in both the American Heart Association (AHA) scientific statement and the European Society of Cardiology (ESC) position paper on SCAD [1, 2]. Regarding SCAD and TS, the authors of the AHA scientific statement on SCAD have concluded that “Care must be taken to ensure that SCAD is not misinterpreted as among others TS” [1]. ESC position paper on SCAD [2] has not either commented on the occurrence of SCAD and TS concurrently; they have only referred to “Takotsubo cardiomyopathy” as one of the differential diagnoses of SCAD [2, 8].

**Fig. 3.**
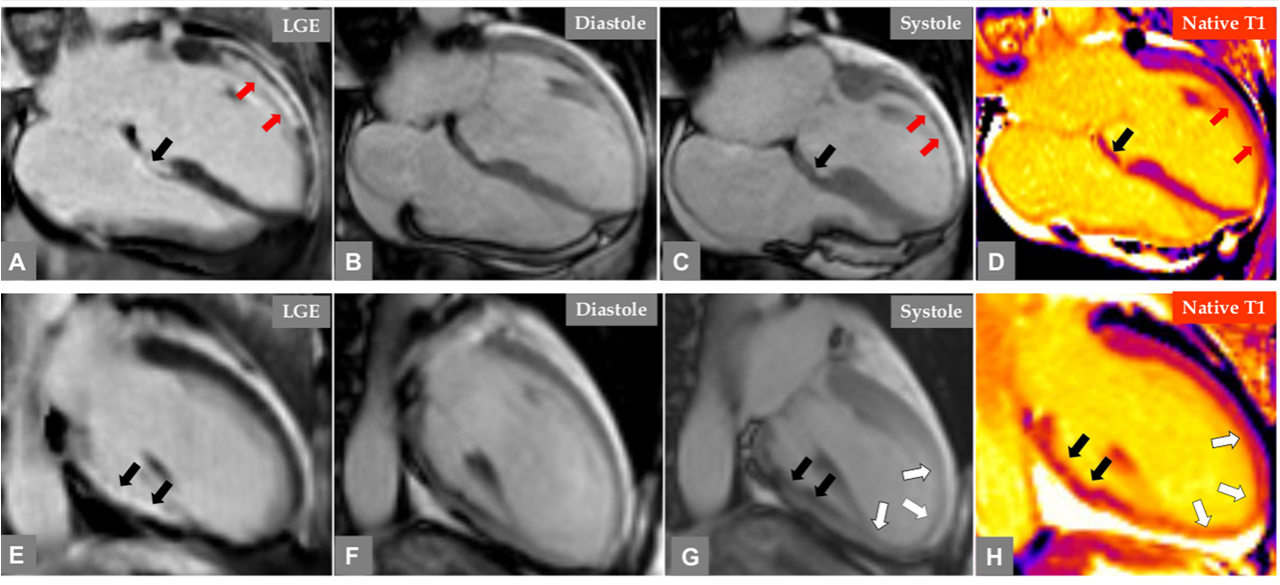
**Cardiac magnetic resonance (CMR) imaging of the same patient in Fig. 2 during the same admission**. CMR images in 4 chamber view (A, B, C, and D) and 2 chamber-view (E, F, G, and H). Late gadolinium enhancement (LGE) images (A and E), Cine images at end-diastole (B and F) and at end-systole (C and G), and native T1 mapping (D and H). Subendocardial to transmural LGE is observed at the basal infero-septal, and basal and mid-inferior left ventricular wall (A and E, black arrows). Cine images reveal akinesia in the same segments (C and G black arrows) while T maps show no evidence of high myocardial T1 in the corresponding segments (D and H black arrows), indicating an old myocardial infarction corresponding to the previously dissected right coronary artery territory. On the other hand, the subendocardial LGE in the basal and mid segment of the anterolateral left ventricular wall (A, red arrows) and the corresponding hypokinesia (C, red arrows) with evidence of elevated myocardial T1 (D, red arrows) denoting a region of acute MI supplied by the acutely occluded and dissected OM branch of the left circumflex artery. Furthermore, there is akinesia in the anterior-apical and inferior left ventricular wall (G, white arrows) with evidence of elevated T1 in the corresponding segments (H, white arrows) but no LGE indicating myocardial stunning with mid-apical ballooning pattern consistent with mid-apical Takotsubo syndrome.

**Fig. 4.**
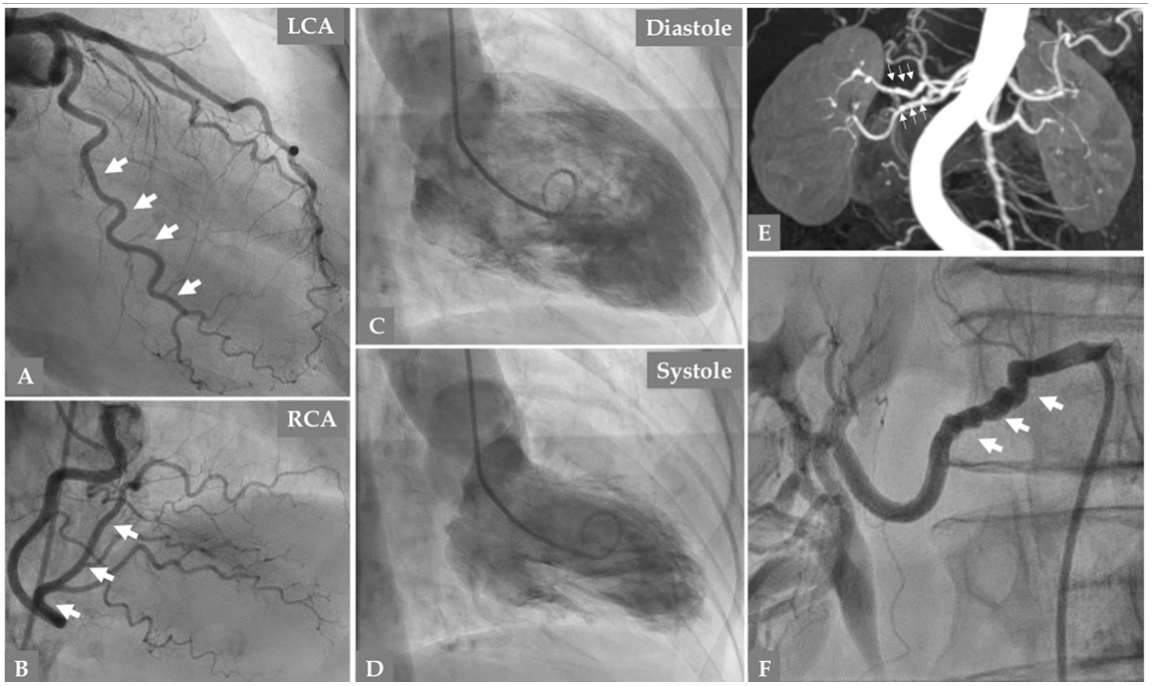
**Follow up coronary angiography 141 days after the second SCAD episode in the same patient in Figs. 1,2,3 reveals complete resolution of the previously dissected large OM branch (A, thick white arrows)**. Normal left main stem and LAD (A). Right coronary artery is normal (B). Contrast Left ventriculography (C, diastole and D, systole) shows normalization of the non-infarcted mid-apical dysfunction confirming the diagnosis of Takotsubo syndrome. Magnetic resonance imaging of the renal arteries (E, thin white arrows) and selective invasive renal artery angiography (F, thick white arrows) reveal signs of fibromuscular dysplasia in the right renal artery (string of beads).

However, the myocardial stunning in SCAD is not restricted to the dissected artery territory, but extended and sometimes extensively so beyond the dissected artery territory and this has been documented in case reports [14], series of case studies [21], in at least two larger studies [22, 43] and even by the same authors who claim that the myocardial stunning is restricted to the dissected artery territory [23]; this have been discussed elsewhere [58, 59, 60]. An example of the association between SCAD and TS has been demonstrated in the illustrative Figures (Figs. 2,3,4). Apart from the LVWMA caused by MI secondary to the old, dissected RCA and the recurrent dissected-OM branch, the cardiac image studies revealed LVWMA in the mid-apical segments with corresponding edema but no late gadolinium enhancement (LGE) and incongruent with the dissected vessels (Figs. 2,3). The mid-apical LVWMA recovered completely during follow up left ventriculography (Fig. 4). These findings were typical for mid-apical TS pattern, which occurred concurrently with the recurrent OM-SCAD demonstrated in Figs. 2,3,4. Furthermore, the improvement of myocardial stunning has nothing to do with the healing of the dissected vessel because myocardial stunning was triggered by ischemia just like any other trigger factor and not caused by ischemia [21, 32, 49].

## 4. Association of SCAD and Fibromuscular Dysplasia

FMD is a non-inflammatory, non-atherosclerotic idiopathic arteriopathy, which most commonly afflicts the renal and internal carotid arteries but may affect almost every arterial bed in the body [65, 66]. The fibrous dysplasia may affect any of the three layers; intima, media, or adventitia of the arterial wall; the most common lesion is the medial fibroplasia (in 70–80% of cases) followed by peri-medial fibroplasia, medial hyperplasia, intimal fibroplasia, and adventitial (periarterial) fibroplasia [1, 65, 66]. FMD usually manifests with stenoses, aneurysms, tortuosity, or dissections. Angiographical morphology of FMD may be multifocal and has typically “string-of-beads” pattern, which is the most common type of FMD, or it may be mono-focal [65, 66]. Renal hypertension due to renal artery involvement and stroke or transient ischemic attack due to carotid or vertebral artery involvement are the most common clinical manifestations of FMD [65].

During the last 10 years, the association of FMD and SCAD has received major attention. Also, the association of SCAD and FMD in the coronary arteries has been described in several case reports during the last 3–4 decades [67, 68, 69]. To our knowledge, the association of coronary FMD and SCAD was described first in 1987 confirmed by cardiac autopsy [67]. SCAD confirmed by CAG and coronary FMD confirmed by cardiac histopathology in the same patient has been reported by Mather *et al*. in 1994 [68]. In 2012, Saw *et al*. [70] reported a series of 6 women with SCAD and concomitant FMD in non-coronary arterial territories such as iliac, renal, and carotid arteries. In one study 2012, signs of FMD were identified incidentally in the iliac artery in 8 (50%) of 16 patients with SCAD during femoral angiogram performed before closure device placement and in the carotid arteries in 2 other patients with carotid artery dissection diagnosed with computed tomography angiography [71]. These 2 patients showed to have FMD in the renal, iliac, and vertebral arteries, with incidental vertebral artery dissection. By screening 50 patients with SCAD for FMD in renal, iliac, and cerebrovascular arteries with computed tomography angiography or magnetic resonance angiography in addition to angiography during the index presentation, Saw *et al*. [72] reported signs of FMD in 43 patients (86%) in ≥1 non-coronary territory. Twenty-five of 43 (58.1%) had signs of FMD in renal arteries; 21 of 43 (48.8%) in iliac arteries, and 20 of 43 (46.5%) in cerebrovascular arteries, six of 43 (14%) had an intracranial aneurysm. However, the prevalence of FMD in patients with SCAD has varied and has been reported in the range of 25% to 86% [3]. In a registry study comprising of 373 SCAD cases, Combaret *et al*. [73] reported FMD at ≥1 arterial site in 45% of cases. The differences in the type of screenings technique and the number of territories screened may explain the variability of the prevalence of FMD in patients with SCAD in different studies.

Coronary involvement of FMD associated with SCAD has also been reported as previously described at autopsy or histopathologic examination [67, 68, 69] or during the last years by invasive CAG [66, 74]. In 2016, Saw and colleagues [66] reported angiographic manifestations suggestive of coronary FMD with several corresponding OCT findings compatible with coronary FMD in a series of 32 patients with confirmed extra-coronary multifocal FMD. Of these 32 patients, 19 presented with MI (13 caused by SCAD) and 13 had stable symptoms.

Systematic screening for FMD in SCAD patients is now recommended by the AHA and the ESC because of the strong association between SCAD and FMD discussed above [1, 2, 3]. This can be done by invasive angiography as renal and iliac artery angiography concurrently with CAG during the index presentation or during repeated follow up angiography. Other imaging modalities are computed tomography angiography specially for extra and intra-cranial arteries, and magnetic resonance imaging for abdominal arteries (Fig. 4E, F).

## 5. Treatment of SCAD

Accurate and instantaneous diagnosis of SCAD is of paramount importance because management of acute MI caused by SCAD differs enormously from that caused by an atherosclerotic process [1, 2]. To select appropriate treatment strategy for the SCAD, the following facts should be known by every interventionalist: first, spontaneous healing of the SCAD lesions occurs in most (73% to 97%) of the cases when repeated CAG is done after 5 weeks [5, 75] (Figs. 1,2,4). Of repeat angiography performed ≥30 days post-SCAD, 152 of 160 (95%) showed spontaneous angiographic healing in a study published by Hassan *et al*. [75]; second, PCI is associated with high technical failure and high complication rates in the form of extension of the IMH in both proximal and distal directions [1, 2, 8, 25, 26]. Because of the underlying arteriopathy in some patients, a new iatrogenic dissection caused by the guide catheter, guide wires and invasive diagnostic catheters such as OCT may occur [1, 2, 8]; third, even if PCI stent implantation is successful, the spontaneous healing of the SCAD lesion may leave the stent unopposed to the vessel wall with the risk for future stent thrombosis [1, 2, 8]; fourth, the same is applied to SCAD patients treated with coronary artery bypass graft (CABG) intervention, the spontaneous healing of SCAD lesions results in future vein graft failure and occlusion [1, 2, 8].

In the absence of ongoing ischemic chest pain, stable clinical condition of the patient, patent dissected coronary artery, and peripheral localization of the SCAD lesions, conservative treatment is the recommended treatment strategy [1, 2, 8]. In a study of 318 SCAD patients, Garcia-Guimaraes [76] reported that conservative management as the initial strategy was adopted in 78% with excellent in-hospital survival, 4 patients (1.3%) died. However, it should be remembered that there is a significant risk of dissection progression or de novo lesions, which may occur in approximately 5–10% of cases during the admission days. For this reason, close monitoring in a cardiac unit for at least 3–5 days is highly recommended [77]. In a pooled analysis of 16 studies (444 patients with SCAD), Bocchino *et al*. [78] reported that most of the major adverse cardiovascular events following SCAD occurred during the in-hospital period with a significant difference compared to the following two semesters, irrespective of the treatment strategy. In a study of 750 SCAD patients, 4% of patients treated conservatively had recurrent MI with 2.5% needing revascularization [63]. In another study by Waterbury and colleagues [25], 41 (17.1%) of 240 SCAD patients underwent emergency revascularization after attempted initial conservative treatment strategy where 8 (4 of them after failed PCI) SCAD patients needed CABG; PCI was performed in the remainder of the cases. Mori *et al*. [79], reported that the SCAD angiographic classification correlates with outcome. At 28 days the adverse composite outcome was higher for the angio-types 2A and 3 with a circumscribed contained IMH and this was maintained during follow-up.

In patients with ongoing symptomatic and electrocardiographic ischemic changes, hemodynamically unstable clinical conditions, lethal arrhythmia, left main stem SCAD and proximal central SCAD lesions, and TIMI 0–1 flow in proximal lesions, PCI should be considered if possible [1, 2, 43]. In certain cases, CABG may be needed as in left main stem SCAD with multivessel SCAD lesions or when PCI has failed. Because of signs of ongoing ischemia or the presence of impaired initial coronary flow, revascularization with PCI was performed in 22% in a study of 318 SCAD patients [43]. In a systematic review of 24 observational studies, Bocchino and colleagues [31] reported on 1720 patients with SCAD comparing a conservative approach with an invasive approach during the period 1990 to 2020. The authors found that the conservative approach was associated with significantly lower target vessel revascularization compared to the invasive strategy. No statistical difference was found regarding all-cause death, cardiovascular death, MI, heart failure, and SCAD recurrence. However, and in general, male gender, smoking habits, diabetes mellitus, prior coronary artery disease, left main coronary artery involvement, lower ejection fraction and low TIMI flow during the index presentation were associated with higher overall mortality.

Consequently, conservative therapy as the initial strategy is adopted in most cases of SCAD and regarded as the pillar treatment strategy. The role of dual antiplatelet in SCAD is not clear. The initiation of acetylsalicylic acid is recommended in the acute stage but the addition of a P2Y12 inhibitor is less clear [1, 2, 3]. In a registry study, Cerrato *et al*. [80] investigated 199 patients in whom SCAD was managed conservatively. They found that, at 1–year follow-up, duel antiplatelet therapy, as compared to single antiplatelet therapy, was independently associated with a higher rate of adverse cardiovascular events, driven by an early excess of non-fatal MI or unplanned PCI. However, patients who undergo PCI with stent implantation should be treated with thrombocyte inhibitors in the conventional way as that in PCI of atherosclerotic lesions [1, 2, 3]. Beta blockers and acetylsalicylic acid are currently adopted as long-term therapy. If the patient was treated conservatively, some investigators recommend addition of clopidogrel during 3 months after the index presentation. Appropriate treatment of hypertension and continuation on beta blockers have shown to diminish SCAD recurrence [27].

## 6. Conclusions

Early and late SCAD recurrences are common complications of SCAD. SCAD may trigger TS. One important predisposing factor for SCAD is the coronary and non-coronary FMD.
